# Glucose-Impaired Corneal Re-Epithelialization Is Promoted by a Novel Derivate of Dimethyl Fumarate

**DOI:** 10.3390/antiox10060831

**Published:** 2021-05-22

**Authors:** Giovanni Giurdanella, Anna Longo, Loredana Salerno, Giuseppe Romeo, Sebastiano Intagliata, Gabriella Lupo, Alfio Distefano, Chiara Bianca Maria Platania, Claudio Bucolo, Giovanni Li Volti, Carmelina Daniela Anfuso, Valeria Pittalà

**Affiliations:** 1 Department of Biomedical and Biotechnological Sciences, Section of Medical Biochemistry, University of Catania, Via S. Sofia 97, 95123 Catania, Italy; g.giurdanella@unict.it (G.G.); LNGNMR86M50G371T@studium.unict.it (A.L.); gabriella.lupo@unict.it (G.L.); alfio.distefano@studium.unict.it (A.D.); livolti@unict.it (G.L.V.); 2 Department of Drug and Health Sciences, University of Catania, 95125 Catania, Italy; l.salerno@unict.it (L.S.); gromeo@unict.it (G.R.); sebastiano.intagliata@unict.it (S.I.); 3 Center for Research in Ocular Pharmacology–CERFO, University of Catania, 95125 Catania, Italy; bucocla@unict.it; 4 Department of Biomedical and Biotechnological Sciences, Section of Pharmacology, University of Catania, Via S. Sofia 97, 95123 Catania, Italy; chiara.platania@unict.it

**Keywords:** SIRC, corneal epithelial cells, high glucose, Nrf2, wound healing, dimethyl fumarate, heme oxygenase-1

## Abstract

Glucose induces corneal epithelial dysfunctions characterized by delayed wound repair. Nuclear erythroid 2-related factor 2 (Nrf2) mediates cell protection mechanisms even through the Heme oxygenase-1 (HO-1) up-regulation. Here, we synthesized new HO-1 inducers by modifying dimethyl fumarate (DMF) and used docking studies to select VP13/126 as a promising compound with the best binding energy to Kelch-like ECH-associated protein 1 (keap1), which is the the regulator of Nrf2 nuclear translocation. We verified if VP13/126 protects SIRC cells from hyperglycemia compared to DMF. SIRC were cultured in normal (5 mM) or high glucose (25 mM, HG) in presence of DMF (1–25 μM) or VP13/126 (0.1–5 μM) with or without ERK1/2 inhibitor PD98059 (15 μM). VP13/126 was more effective than DMF in the prevention of HG-induced reduction of cell viability and proliferation. Reduction of wound closure induced by HG was similarly counteracted by 1 μM VP13/126 and 10 μM DMF. VP13/126 strongly increased phospho/total ERK1/2 and restored HO-1 protein in HG-treated SIRC; these effects are completely counteracted by PD98059. Moreover, high-content screening analysis showed a higher rate of Nrf2 nuclear translocation induced by VP13/126 than DMF in HG-stimulated SIRC. These data indicate that VP13/126 exerts remarkable pro-survival properties in HG-stimulated SIRC, promoting the Nrf2/HO-1 axis.

## 1. Introduction

Corneal epithelium represents a multilayered cellular barrier that lines the ocular surface [1]. It constitutes a proper cellular barrier of the innate immune system against mechanical stimuli, microbial invasion and environmental insults [2]. Corneal epithelial dysfunction is observed in concurrent inflammation conditions or ocular surface disease, producing a delay of corneal re-epithelialization [3,4]. Age-related eye diseases are often associated to impairment of the corneal epithelium, increasing the risk of bacterial infections and compromising the refractive eye’s capabilities [5]. Diabetes causes profound ocular changes by altering the eye barriers. It has been shown that the exposure of retinal microcapillary endothelial cells and pericytes to high glucose concentrations leads to the breaking of the retinal blood barrier [6,7]. Furthermore, diabetic patients show enhanced incidence of corneal disease, such as diabetic keratopathy, characterized by epithelial scarring defects, infections, ulcers and permanent vision loss [8,9,10,11]. Diabetic condition elicits a chronic oxidative stress and an inflammatory status that supports the progressive cellular dysfunction and the alterations of physiological processes of the eye [12]. High concentration of glucose was shown to compromise corneal barrier function without an associated decrease in the levels of zonula occludens proteins (ZO-1, ZO-2, ZO-3), tight junction proteins (claudin-1) and occludin [13]. Such an effect is due to the loss of laminin-5, one of the main constituents of corneal basement membrane, under a HG condition: clinical features of diabetic keratopathy were shown to correlate with decreased adhesion between epithelial cells [14]. In vitro data indicate that HG produce a delayed corneal wound healing and cell migration as consequence of increased reactive oxygen species (ROS) production and impairment of both AKT (also called protein kinase B or PKB) signaling and cell survival [15,16,17].

The nuclear erythroid 2 related factor 2 (Nrf2), encoded by Nfe2l2 gene, regulates the redox homeostasis in mammals activating the cellular protective antioxidant response and phase II detoxification enzymes [18,19]. Nrf2 promotes transcription of genes such as heme oxygenase-1 (HMOX1), thioredoxin reductase 1 (Txnrd1), glutamate-cysteine ligase (GCL) and NAD(P)H-quinone oxidoreductase 1 (NQO1) through the interaction with the antioxidant response elements (AREs), whose consensus sequences are present in their regulatory elements [20]. Nrf2-mediated activation of cellular functions produces a homeostatic response that represents an evolutionarily conserved mechanism providing an endogenous cellular protection against oxidative damage [21]. It was shown that the activation of Nrf2 induces HO-1-mediated production of the anti-inflammatory compounds, carbon monoxide (CO) and bilirubin and inhibits the activation of the nuclear factor kappa-light-chain-enhancer of activated B cells (NF-ĸB) enhancing IKKβ degradation [22,23]. The pivotal role of Nrf2 as a “switch” of the cellular response against oxidative damage places new emphasis regarding the study of new molecular strategies that mediate its activation [24,25].

Dimethyl fumarate (DMF) is known to prevent pro-oxidant insults through the extracellular signal-regulated kinase ERK1/2-dependent activation of Nrf2/HO-1 pathway, restoring the functions of mitochondria in different models such as cardiomyocytes [26], retinal pigment epithelium (RPE) [27] and retinal ganglion cells [28]. Moreover, when tested in diabetic conditions, DMF ameliorated epithelial wound healing and reduced both oxidative damage and inflammation in vitro and in vivo, producing the activation of Nrf2 signaling [29,30]. However, DMF was shown to produce a controversial effect considering that induced Nrf2 nuclear translocation similarly in normal or HG cultured retinal pigment epithelium, without a significant enhancement of wound healing [31]. On the other hand, recent findings reported Nrf2/HO-1 signaling pathway as a promising target for the corneal epithelial cell protection from oxidative stress and regeneration [32,33,34,35]. HO-1 induction through Nrf2 translocation is regulated by covalent binding to the Kelch-like ECH-associated protein 1 (Keap1), which is the regulator of Nrf2 nuclear translocation. Covalent binding to Keap1 occurs to electron poor antioxidant compounds, through Michael addition to double bonds of these compounds [36]. Thus, new efforts are plausible to improve molecular strategies that enhance the cellular response against oxidative stress. In the present study, we aimed to identify a new inducer of the antioxidant response and Nrf2/HO-1 pathway through the chemical modification of the well-known DMF. Molecular docking and Molecular-Mechanics Generalized Born Surface Area (MM-GBSA) calculations helped to identify VP13/126 (compound **4**) as the best HO-1 inducer, compared to the other compounds of a small congeneric series composed by also VP11/100 (compound **1**), VP12/72 (compound **2**) and VP11/102 (compound **3**). As it has been demonstrated that DMF derivatives are good HO-1 expression/activation inducers [37], we analyzed the putative role of this new compound VP13/126 to protect SIRC from cell damage induced by HG damage. In particular, we tested the capability of VP13/126 to counteract the reduction in cell viability and wound healing induced by glucose toxicity and the underlying molecular mechanisms activated by this compound. DMF activity in Nrf2/HO-1 activation was used as positive control.

## 2. Materials and Methods

### 2.1. Chemistry

Reactions were followed by thin layer chromatography performed on Sigma Aldrich silica plates (60 F254), visualized by UV light (readings at 254 nm and 366 nm) and/or using I_2_ chamber. Purification by flash chromatography was achieved by using Merck silica gel 60, 0.040‒0.063 mm (230‒400 mesh) as stationary phase. Melting points were measured by meas of an IA9200 Electrothermal apparatus furnished with a digital thermometer in capillary glass tubes and are uncorrected. Infrared spectra (IR) were registered using KBr disks or NaCl crystal windows. ^1^H NMR spectra were registered on a Varian Unity Inova 200 MHz spectrometer in DMSO-*d*_6_ solution. Chemical shifts are given in δ values (ppm), using tetramethylsilane (TMS) as the internal standard; coupling constants (*J*) are given in Hz. Signal multiplicities are reported as s (singlet), d (doublet), t (triplet), q (quartet) or m (multiplet). Elemental analyses for C and H were within ±0.4% of theoretical values and were determined through a Carlo Erba Elemental Analyzer Mod. 1108 apparatus. Reactants and solvents were bought from commercial vendors.

#### 2.1.1. General Procedure for the Synthesis of (E)-4-oxo-4-Phenylbut-2-Enoic Acids (1–2)

In a round bottom flask, maleic anhydride (5 mmol, 1 eq) was combined with benzene or toluene (2 mL). To the resulting suspension was carefully added AlCl_3_ (10 mmol, 2 eq) portion-wise. The suspension was stirred for 2 h. The reaction was stopped with crushed ice and HCl 37% (1:1) and the reaction solvent was removed under reduced pressure. The resulting solid was triturated with Na_2_CO_3_ 5% (20 mL); the suspension was filtered, and the filtrate was acidified with HCl 37%. The obtained precipitate was filtered under reduced pressure and finally triturated with cyclohexane.

#### 2.1.2. (E)-4-Oxo-4-phenylbut-2-enoic acid (1)

Whitish solid (57%): mp 92–93 °C; IR (KBr) cm^−1^ 1703, 1672, 1409, 1332, 1302, 1191, 730; ^1^H NMR (200 MHz, DMSO-*d*_6_) δ 7.96 (d, *J* = 8.4 Hz, 2H, aromatic), 7.89 (d, *J* = 15.5 Hz, 1H, COC*H*=CHCOOH), 7.76–7.69 (m, 1H, aromatic), 7.63–7.55 (m, 2H, aromatic), 6.69 (d, *J* = 15.5 Hz, 1H, COCH=C*H*COOH).

#### 2.1.3. (E)-4-Oxo-4-(4-methylphenyl)but-2-enoic acid (2)

Yellowish solid (54%): mp 138–139 °C; IR (KBr) cm^−1^ 1700, 1662, 1602, 1409, 1238, 1300, 1186, 1012, 983, 758; ^1^H NMR (200 MHz, DMSO-*d*_6_) δ 7.97–7.85 (m, 2H + 1H, aromatic + COC*H*=CHCOOH), 7.39 (d, *J* = 8.2 Hz, 2H, aromatic), 6.67 (d, *J* = 15.6 Hz, 1H, COCH=C*H*COOH), 2.41 (s, 3H, CH_3_).

#### 2.1.4. General Procedure for the Synthesis of Methyl (E)-4-oxo-4-Phenylbut-2-Enoates (3–4)

In a round bottom flask compound **1** or **2** (1 mmol, 1 eq) was solubilized in 5 mL of methanol and 2 drops of concentrated H_2_SO_4_ were added. The solution was refluxed for 2 h. The cooled solution was concentrated under vacuo and the crude was diluted with EtOAc (120 mL). The organic phase was washed with NaHCO_3_ 5% (2 × 60 mL) and brine (1 × 60 mL); the organic phase was treated with Na_2_SO_4_, filtered and concentrated under reduced pressure. The obtained mixture was purified by flash chromatography eluting with a mixture of Cy/EtOAc (9:1).

#### 2.1.5. Methyl (E)-4-oxo-4-Phenylbut-2-Enoate (3)

Yellowish solid (29%): mp 29–30 °C; IR (KBr) cm^−1^ 1715, 1665, 1620, 1590, 1570, 975; ^1^H NMR (200 MHz, DMSO-*d*_6_) δ 8.07–7.94 (m, 3H, aromatic + COCH=CHCOOCH_3_), 7.77–7.69 (m, 1H, aromatic), 7.62–7.55 (m, 2H, aromatic), 6.76 (d, *J* = 15.6 Hz, 1H, COCH=CHCOOCH_3_), 3.79 (s, 3H, CH_3_). Anal. calcd. for C_11_H_10_O_3_: C, 69.46; H, 5.30, found: C, 69.22; H, 5.29.

#### 2.1.6. Methyl (E)-4-oxo-4-(4-Methylphenyl)but-2-Enoate (4) (VP13/126)

Yellow solid (48%): mp 46–47 °C; IR (KBr) cm^−1^ 1727, 1674, 1602, 1314, 1170, 976, 754; 1H NMR (200 MHz, DMSO-*d*_6_) δ 8.01–7.93 (m, 2H + 1H, aromatic + COCH=CHCOOCH_3_), 7.40 (d, *J* = 8.2 Hz, 2H, aromatic), 6.74 (d, *J* = 15.4 Hz, 1H, COCH=CHCOOCH_3_), 3.79 (s, 3H, COOCH_3_), 2.41 (s, 3H, CH_3_). Anal. calcd. for C_12_H_12_O_3_: C, 70.58; H, 5.92, found: C, 70.41; H, 5.93.

#### 2.1.7. Synthesis of Ethyl 4-oxo-4-(4-Methylphenyl)Butanoate (5)

In a round bottom flask, 4-(4-methylphenyl)-4-oxobutyric acid (5 mmol, 1 eq) was solubilized in 10 mL of ethanol. To the obtained solution were added 100 μL of concentrated H_2_SO_4_ and the mixture was refluxed for 1 h. Then, a saturated solution of Na_2_CO_3_ (80 mL) was added to the mixture and extracted with CH_2_Cl_2_ (3 × 80 mL); the reunited organic phases were washed with brine, dried with Na_2_SO_4_, filtered and concentrated under reduced pressure. The product thus obtained did not require any further purification. White solid (80%): mp 41–42 °C; IR (KBr) cm^−1^ 1724, 1678, 1608, 1477, 1416, 1375, 1227, 1162, 1066, 1031, 820; ^1^H NMR (200 MHz, DMSO-*d*_6_) δ 7.89 (d, *J* = 8.2 Hz, 2H, aromatic), 7.34 (d, *J* = 8.0 Hz, 2H, aromatic), 4.05 (q, *J* = 7.4 Hz, 2H, CH_2_CH_3_), 3.27 (t, *J* = 6.0 Hz, 2H, COCH_2_CH_2_), 2.62 (t, *J* = 6.0 Hz, 2H, COCH_2_CH_2_), 2.38 (s, 3H, CH_3_), 1.16 (t, *J* = 7.2 Hz, 3H, CH_2_CH_3_).

#### 2.1.8. Synthesis of Ethyl (E)-4-Oxo-4-(4-Methylphenyl)but-2-Enoate (6)

Ethyl 4-oxo-4-(4-methylphenyl)butanoate (**5**) (2.27 mmol, 1 eq) was dissolved in 5 mL of dry CH_2_Cl_2_ under N_2_ atmosphere and under stirring. To the resulting solution were dropped a solution of Br_2_ (2.5 mmol, 1.1 eq) in 1.3 mL of anhydrous CH_2_Cl_2_ and 5 drops of concentrated HCl as catalyst at room temperature within 1 h. After this period of time, the temperature was lowered to 0 °C with an ice bath and 1 mL of TEA was dropped under N_2_ within 0.5 h and the reaction was left stirring for an additional time of 0.5 h. The crude was diluted with 100 mL of CH_2_Cl_2_ and washed in sequence with deionized H_2_O (2 × 150 mL), HCl 1N (2 × 150 mL), Na_2_CO_3_ 5% (2 × 150 mL) and brine (1 × 150 mL). The organic phase was dried with Na_2_SO_4_, filtered and evaporated. The mixture was purified by flash chromatography eluting with a mixture of Cy/EtOAc (9:1). Yellow oil (50%): IR (NaCl) cm^−1^ 1723, 1670, 1606, 1299, 1270, 1168, 1038, 1011, 974, 878, 825, 757; ^1^H NMR (200 MHz, DMSO-*d*_6_) δ 8.00–7.91 (m, 2H + 1H, aromatic + COCH=CHCOO), 7.40 (d, *J* = 7.8 Hz, 2H, aromatic), 6.72 (d, *J* = 15.0 Hz, 1H, COCH=CHCOO), 4.25 (q, *J* = 6.6 Hz, 2H, CH_2_CH_3_), 2.41 (s, 3H, CH_3_), 1.28 (t, *J* = 6.6 Hz, 3H, CH_2_CH_3_). Anal. calcd. for C_13_H_14_O_3_: C, 71.54; H, 6.47, found: C, 71.33; H, 6.45.

#### 2.1.9. Synthesis of Phenethyl 4-oxo-4-(4-Methylphenyl)Butanoate (7)

In a round bottom flask, 4-(4-methylphenyl)-4-oxobutyric acid (5 mmol, 1 eq) was dissolved in 12 mL of DMSO. Na_2_CO_3_ (6 mmol, 1.2 eq) was added and the mixture stirred at 22 °C for 0.5 h. Then, phenethyl bromide (5.65 mmol, 1.13 eq) dissolved in 2 mL of DMSO was dropped to the solution at 22 °C within 0.5 h. Once the addition was completed, a spatula tip of KI was added, and the reaction was stirred at 22 °C for 24 h. The mixture was diluted with EtOAc (100 mL) and washed with HCl 1N (2 × 150 mL), NaHCO_3_ (2 × 150 mL) and brine (1 × 150 mL). The organic phase was dried with Na_2_SO_4_, filtered and evaporated. The product was obtained as a pure solid and was directly used in the next step with no further purification. White solid (72%): mp 66–67 °C; IR (KBr) cm^−1^ 1725, 1676, 1608, 1414, 1390, 1356, 1226, 1160, 987, 825; ^1^H NMR (200 MHz, DMSO-*d*_6_) δ 7.88 (d, *J* = 8.2 Hz, 2H, aromatic), 7.36–7.20 (m, 7H, aromatic), 4.21 (t, *J* = 6.8 Hz, 2H, COOCH_2_CH_2_), 3.25 (t, *J* = 5.8 Hz, 2H, COCH_2_CH_2_), 2.87 (t, *J* = 6.8 Hz, 2H, COOCH_2_CH_2_), 2.58 (t, *J* = 5.8 Hz, 2H, COCH_2_CH_2_), 2.39 (s, 3H, CH_3_).

#### 2.1.10. Synthesis of Phenethyl (E)-4-oxo-4-(4-Methylphenyl)but-2-Enoate (8)

Phenethyl 4-oxo-4-(4-methylphenyl)butanoate (**7**) (1.35 mmol, 1 eq) was dissolved in 4 mL of dry CH_2_Cl_2_ under N_2_ atmosphere and under stirring. To the resulting solution were dropped a solution of Br_2_ (1.49 mmol, 1.1 eq) in 0.763 mL of dry CH_2_Cl_2_ and 5 drops of concentrated HCl as catalyst at room temperature within 1 h, the temperature was lowered to 0 °C with an ice bath and 1 mL of TEA was dropped under N_2_ within 0.5 h and the mixture was left stirring for an additional time of 0.5 h. The mixture was diluted with 100 mL of CH_2_Cl_2_ and washed in sequence with deionized H_2_O (2 × 150 mL), HCl 1N (2 × 150 mL), Na_2_CO_3_ 5% (2 × 150 mL) and brine (1 × 150 mL). The organic phase was dried with Na_2_SO_4_, filtered and evaporated. The crude was purified by flash chromatography eluting with a mixture of Cy/EtOAc (9:1). Deliquescent yellow solid (82%): IR (NaCl) cm^−1^ 1719, 1670, 1604, 1301, 1165, 975, 755, 700; ^1^H NMR (200 MHz, DMSO-*d*_6_) δ 7.96–7.88 (m, 2H + 1H, aromatic + COCH=CHCOO), 7.40 (d, *J* = 8.0 Hz, 2H, aromatic), 7.34–7.19 (m, 5H, aromatic), 6.69 (d, *J* = 15.6 Hz, 1H, COCH=CHCOO), 4.41 (t, *J* = 6.8 Hz, 2H, COOCH_2_CH_2_), 2.99 (t, *J* = 6.8 Hz, 2H, COOCH_2_CH_2_), 2.41 (s, 3H, CH_3_). Anal. calcd. for C_19_H_18_O_3_: C, 77.53; H, 6.16, found: C, 77.28; H, 6.17.

### 2.2. Molecular Docking

VP13/126, compounds **1**–**4**, **6**, **8**, and DMF structure files were built with the “Online SMILES Translator and Structure File Generator” https://cactus.nci.nih.gov/translate/ (accessed on 15 December 2020). Tridimensional structures of compounds, ionization state at pH 7.4, were built with the LigPrep task of Schrödinger Maestro. The full-length structure of KEAP1 was built by means of the Advanced Molecular Modeling Task of Schrödinger Maestro, as described previously [38]. Covalent docking simulation within the Schrödinger Maestro suite was carried out with the CovDock task, setting as reactive residue Cys151 and reaction simulation the Michael addition to double bonds. Molecular Mechanics/Generalized Born Surface Area (MM-GBSA) rescoring of complex was carried out with Prime calculations within the Schrödinger Maestro package, applying an implicit solvation model of the system and allowing structure minimization of all residues within 15 Å from ligands.

### 2.3. Reagents

Dimethyl fumarate (DMF) was provided by Sigma Aldrich Co. (St. Louis, MO, USA). PD98059 was provided by Calbiochem (La Jolla, CA, USA). Rabbit polyclonal antibody against phospho-p44/42 MAPK (p-ERK1/2, catalog n. 9101), p44/42 MAPK (ERK1/2, catalog n. 9102), phospho-AKT (catalog n. 4060S), AKT (catalog n. 9272S) and GAPDH (catalog n. 2118) were provided by Cell Signaling Technology (Danvers, MA, USA); rabbit polyclonal antibody against Nrf2 (catalog n. ab137550) was provided by Abcam (Cambridge, UK). All reagents used for cell cultures and stripping buffer were purchased from Invitrogen Thermo Fisher Scientific (Monza, Italy).

### 2.4. SIRC Culture

Statens Seruminstitut rabbit corneal (SIRC) epithelial cells (ATCC CCL-60) were cultured in Eagle’s Minimum Essential Medium (ATCC^®^ 30–2003TM) supplemented with 10% activated fetal bovine serum (FBS, 10108–165, GIBCO), Earle’s salts, L-glutamine, and non-essential amino acids. Cells were maintained in a humidified atmosphere of 5% CO_2_ at 37 °C. SIRC were cultured in serum-free EMEM, for 24, 48 and 72 h with 5 mM of glucose considered as control condition (normal glucose, NG), or treated with 25 mM of glucose (high glucose, HG) or with 25 mM of mannitol. VP13/126 (at concentration of 0.1, 1 and 5 µM) or dimethyl fumarates, DMF (at concentration of 1, 10 and 25 µM) were included in the control condition (NG) and in HG medium for 24, 48 and 72 h. Treatments were carried out upon reaching about 70% of cell confluence.

### 2.5. Analysis of Cell Viability

Cell viability was evaluated by MTT assays after treatments, as previously reported [39]. 2 × 10^4^ cells/well of SIRC were plated in 96-well plates and incubated overnight at 37 °C. Then, 0.1, 1 and 5 μM of VP13/126 and 1, 10 and 25 μM of DMF were added to each well with normal glucose (NG) or with high glucose (HG) in a treatment volume of 200 μL. After incubation for 24, 48 and 72 h, we added 10 μL of MTT [3-(4,5-dimethylthiazol-2-yl)2,5-diphenyltetrazolium bromide] reagent (5 mg/mL) to each well allowing the formazan crystals formation for 3 h. Subsequently, were added 100 μL of DMSO per well and measured the absorbance at 570 nm with plate reader (Biotek Instruments, Elx-800). Obtained values were reported in graphs as a percentage of the control.

### 2.6. Wound Healing Assay

The putative involvement of treatments on the epithelial migration were evaluated in SIRC by a standard wound healing assay [40]. Briefly, confluent cell monolayers were wounded by scratching with a P200 pipette tip, then were washed with PBS 1X and finally were incubated with medium containing the treatments (at time 0). Wound area was analyzed from six different wells for each treatment, performed in tree independent cell cultures. Wound closure was monitored by acquiring photographs at 40× magnification using a bright-field microscope at each time point of 0, 24, 48 and 72 h from randomly selected fields. Image J software (Broken Symmetry Software, Bethesda, MD, USA) was used to analyzed SIRC wound closure by evaluating the migration of the cellular front over time into the cell-free wound area.

### 2.7. Proliferation Assay

SIRC proliferation was analyzed by crystal violet assays as previously described [41]. At the end of the treatment, the cells were washed with PBS 1X and subsequently were both fixed and stained with 0.5% crystal violet solution in 20% methanol for 10 min. Then, the cells were washed with water and dried. Measurement of trypan blue staining was carried out by assessing the absorbance at 570 nm with the microplate reader (Synergy 2-BioTek).

### 2.8. High-Content Screening (HCS) and Image Analysis

High-content screening was carried out to assess the extent and the nuclear translocation of Nrf2 transcription factor in SIRC [42]. Cells were seeded in 12-well and treated in NG or in HG with or without 5 μM of VP13/126, or 15 μM of PD98059 for 48 h (described in Section 2.3.) After the treatment, SIRC were washed three times with PBS 1X and were fixed with 4% paraformaldehyde for 0.5 h at 4 °C. Then, cells were incubated for 30 min in a 5% solution normal goat serum and 0.2% Triton X in PBS in order to perform permeabilization and blocking. Protein staining was carried out by incubated cells overnight at 4 °C with primary antibody against Nrf2 in a PBS/triton 0.1% solution. Primary antibody was tested at 1:150 of dilution. Excess of primary antibody was removed washing cells tree times with 0.2% Tween20 in PBS solution. Then, we performed the incubation with the Alexa Fluor 488 conjugated secondary antibody in PBS (1:200 of concentration) for 1 h, at room temperature. Subsequently, secondary antibody was removed and after cells washing three time in PBS 1X, we stained nuclei with NucBlue solution for 15 min at room temperature as described in the datasheet provider (Thermo Fisher Scientific, Monza, Italy). Images were acquired using the PerkinElmer Operetta High-Content Imaging System (# HH12000000). Plates were analyzed in confocal conditions using the 20× long WD objective. We acquired images using UV light, em: 460 nm, channel for NucBlue stained nuclei, shown in blue and Alexa Fluor 488 channel (ex: 496 nm, em: 519 nm) for Nrf2 staining, shown in green. Analysis was carried out by using at least 200 cells captured per well. Harmony high-content imaging and analysis software (PerkinElmer) was used to perform the analysis of all images. First of all, the software carried out cell segmentation in the DAPI channel by identifying blue stained nuclei by screening with Area > 30 µm. Then, cytoplasm was identified in segmented cell based on green staining. Mean intensity per well of Alexa Fluor 488 in cytoplasm was considered as Nrf2 protein levels using the Calculate Intensity Properties function. Nuclear translocation of Nrf2, was evaluated after nuclei identification, we considered the fluorescence intensity of Alexa Fluor 488 (as indicative of Nrf2 protein levels) into the nuclei population of the control SIRC using Select Population function. Then, we identified the threshold level of green fluorescence intensity up to 95° percentile of the nuclei population in untreated SIRC. Nuclear translocation of the different experimental conditions was calculated by considering as positive (i.e., translocated) all nuclei with greater intensity for Alexa Fluor 488 staining than the threshold value. Finally, the percentage of positive nuclei for Nrf2 was calculated using Calculate Properties function using the formula: A/B × 100, where A was the number of positive nuclei and B was the number of analyzed objects (cells). Final output values of the nuclear translocation evaluation were reported as mean per well.

### 2.9. Western Blot Analysis

Western blot was used to analyze proteins extracted from whole cell lysates as described previously [43]. Cell lysates were produced using RIPA buffer (supplemented with protease and phosphatase inhibitor cocktails) (Sigma-Aldrich, St. Louis, MO, USA). Protein amounts of 30 µg were loaded on 4–20% precast polyacrylamide gel (Mini-PROTEAN^®^ TGX™ Precast Protein Gels, (Bio-Rad Laboratories, Milan, Italy) and separated by electrophoresis. Then, proteins were transferred to nitrocellulose membranes. Subsequently, membranes were blocked for 0.5 h with Odyssey Blocking Buffer (LI-COR Biosciences, Lincoln, NE, USA), and were incubated at 4 °C overnight with the antibodies against phospho-ERK 1/2 (1:500), total ERK 1/2 (1:1000), HO-1 (1:2000), phospho-AKT (1:500), total AKT (1:800) and Nrf2 (1:1000). GAPDH (1:1000) served as the loading control. Immunoblot was detected through Odyssey Imaging System (LI-COR Biosciences, Lincoln, NE, USA). Densitometry analyses of blots were performed using the Image J software.

### 2.10. Statistical Analysis

Each experiment was carried out four times (*n* = 4), i.e., MTT, Crystal violet, WB and wound healing assay. Experiments were performed in triplicate for each cell culture plate and Petri dish. Data are reported as mean ± SEM. The different groups/conditions were compared by one-way or two-way analysis of variance (ANOVA) and Tukey–Kramer post hoc test; a *p* value < 0.05 was considered to denote a statistically significant difference between experimental and control group. Statistical analysis and graph design were carried out by means of GraphPad Prism 7 software (GraphPad Inc., San Diego, CA, USA).

## 3. Results

### 3.1. Chemistry

**Scheme 1:**
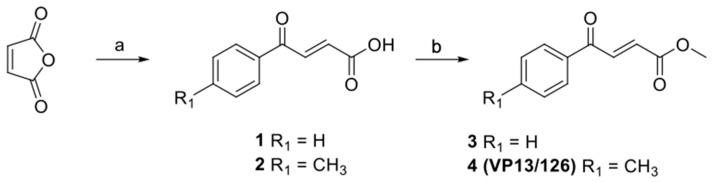
Reagents and conditions: (**a**) benzene or toluene, AlCl_3_, room temperature, 2 h; (**b**) CH_3_OH, H_2_SO_4_, reflux, 2 h.

Scheme 1 reports the synthesis of monomethyl fumarates 3–4. Briefly, intermediates 1–2 were obtained through a Friedel–Craft acylation of benzene or toluene with maleic anhydride and AlCl_3_ at room temperature for 2 h; then, final compounds **3** and **4** (VP13/126) were obtained through Fisher esterification with refluxing methanol and concentrated H_2_SO_4_ for 2 h.

**Scheme 2:**
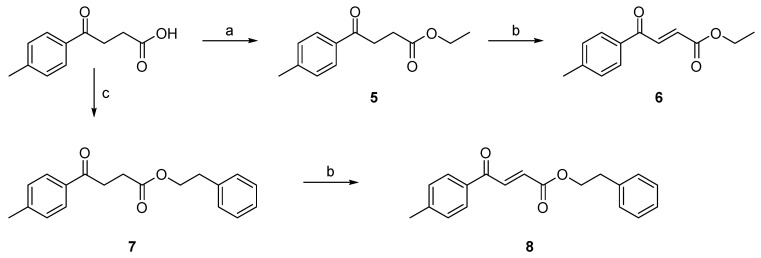
Reagents and conditions: (**a**) EtOH, H_2_SO_4_, reflux, 1 h; (**b**) Br_2_, anhydrous CH_2_Cl_2_, HCl concentration, room temperature, 1 h, then TEA, 0 °C, 0.5 h; (**c**) phenethyl bromide, KI, DMSO, room temperature, 24 h.

In Scheme 2 is depicted the synthesis of compounds **6** and **8**. The final compounds has been obtained using 4-(4-methylphenyl)-4-oxobutyric acid as starting material. Specifically, a Fisher esterification with refluxing ethanol and concentrated H_2_SO_4_ afforded intermediate **5** which has been converted into its α,β-unsaturated derivative **6** by treatment with bromine in anhydrous CH_2_Cl_2_ and TEA as base.

### 3.2. Covalent Molecular Docking and Affinity to Keap1 (MM-GBSA Calculations)

**Figure 1:**
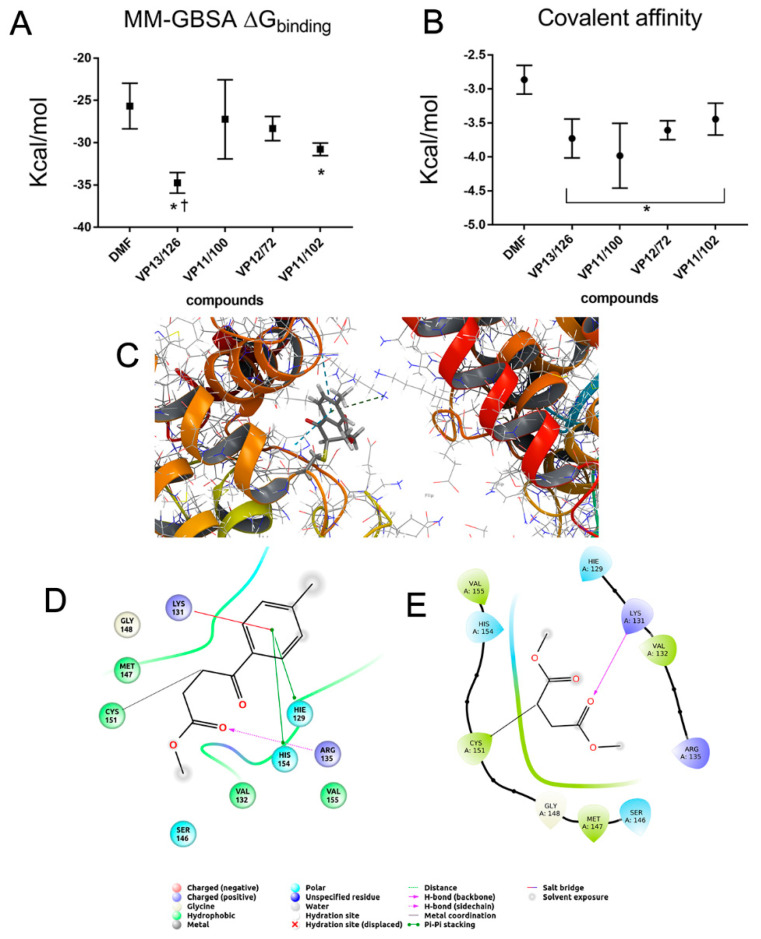
VP13/126 has the best (more negative) predicted ΔG_binding_ energy for Keap1. (**A**) Binding free energy of compounds covalently docked to Keap, binding free energy was predicted through MM-GBSA calculation. (**B**) Covalent docking affinity as determined from covalent docking simulation. (**C**) 3D representation of VP13/126 pose bound covalently to Cys151 of Keap1 homodimer. (**D**) 2D representation of VP13/126 interactions with Keap1. (**E**) 2D representation of DMF interactions with Keap1. * *p* < 0.05 vs. DMF; † *p* < 0.05 vs. VP11/102 (compound **3**); *n* = 4.

Upon binding, through Michael addition, to cysteine residues of Keap1 homodimer the complex oligomerize promoting Nrf2 translocation to the nucleus. We simulated covalent docking using as reaction site Cys-151 [44]. The compounds of the small congeneric series of molecules reported in Scheme 2. Compounds 6 and 8 did not passed the first step of covalent molecular docking due to high not favorable Coulomb energy c, which negatively influenced the covalent affinity of these two compounds, that was lower than DMF. Covalent affinity values of compounds **6** and **8** were, respectively, 1.5 ± 0.6 and 1 ± 0.5. After covalent docking, MM-GBSA calculations were carried out for compounds VP11/100 (compound **1**), VP12/72 (compound **2**), VP11/102 (compound **3**) and VP13/126 (compound **4**). VP13/126 showed significant more negative binding free energy (ΔG_binding_), than DMF and other VP compounds (Figure 1A). Furthermore, all VP compounds showed better covalent affinity compared to DMF. The better ΔG_binding_ energy (more negative), associated to VP13/126-Keap1 complex, could be accounted to greater number of interactions π-stacking and cation-π (Figure 1C,D) of the compound, compared to the other analyzed, then leading to increased electrophile characteristics of VP13/126 and increased the Michael Addition rate. On the contrary, the pose of DMF covalently bound to Keep shows only a H-bond between a carbonylic oxygen and Lys131 (Figure 1E).

### 3.3. VP13/126 Prevented HG-Induced Reduction of Cell Viability in SIRC

**Figure 2:**
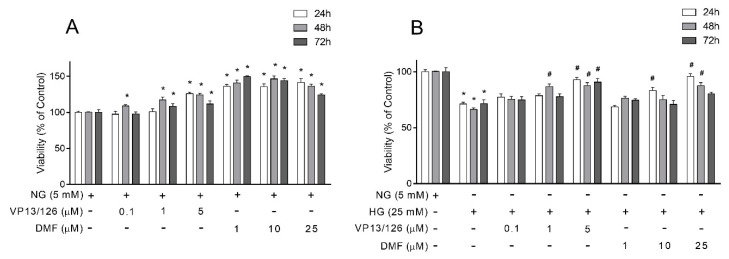
Effects of VP13/126 and DMF on cell viability of SIRC stimulated by HG. (A): MTT assays were carried out in cells treated for 24, 48 and 72 h with normal glucose (5 mM) with or without increasing amounts of VP13/126 (0.1, 1 and 5 µM) or DMF (1, 10 and 25 µM). (B): cells treated with HG (25 mM) with or without increasing amounts of VP13/126 (0.1, 1 and 5 µM) or DMF (1, 10 and 25 µM). Values are expressed as a mean ± SEM of three independent experiments, each run in triplicate. * *p* < 0.05 vs. NG; # *p* < 0.05 vs. HG alone in the respective conditions. Two-way ANOVA, followed by Tukey’s test.

In order to evaluate the effects of VP13/126 on cell viability (mitochondrial function), we performed MTT assays after treatments of SIRC with increasing concentrations of VP13/126 (0.1, 1 and 5 μM) in comparison to DMF treatments (at concentration of 1, 10 and 25 μM) for 24, 48 and 72 h. As shown in Figure 2, panel A, VP13/126 slightly increased cell viability in dose and time-dependent manner, producing a maximum increased level of about 15 and 25% at the concentrations of 1 and 5 μM, respectively.

A more robust effect was observed in DMF-treated SIRC with an increase of about 50% in cell viability at concentration of 1 μM for 72 h of treatment. To mimic in vitro the insult to corneal epithelium occurring in diabetes, we growth SIRC in high concentrations of glucose (25 mM, HG). Moreover, MTT assays showed that exposure to HG at 24, 48 and 72 h reduced by about 30% viability of SIRC compared to NG-treated cells (Ctrl). Such a reduction in cell viability was not observed in HM-treated SIRC (data not shown). Co-treatments with increasing concentrations of VP13/126, triggered a significant restore in cell viability at concentration of 1 and 5 μM. Unlike VP13/126 treatment, DMF showed a milder effect at the 25 μM of concentration producing a significant restore in cell viability only after 24 and 48 h of co-treatment of SIRC with HG. These data indicate the VP13/126 exerts a considerable effect in the prevention of cell viability reduction induced by HG in SIRC.

### 3.4. VP13/126 Stimulated Cell Proliferation in SIRC Challenged with HG

**Figure 3:**
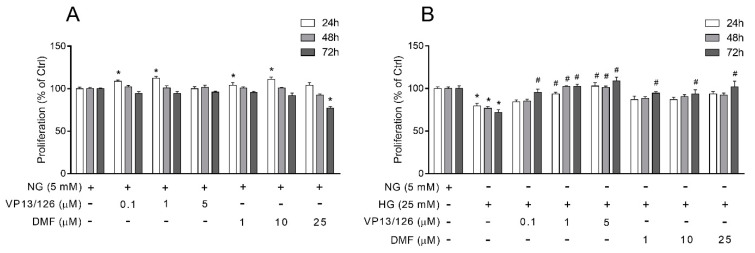
Effects of VP13/126 and DMF restored cell proliferation of SIRC challenged with high glucose. Crystal violet assays were performed in cells cultured for 24, 48 and 72 h with normal glucose (Ctrl, 5 mM) in presence of increasing amounts of VP13/126 (0.1, 1 and 5 µM) or DMF (1, 10 and 25 µM) (**A**). Cells were treated with high glucose (HG, 25 mM) supplemented with increasing amounts of VP13/126 (0.1, 1 and 5 µM) or DMF (1, 10 and 25 µM) were also tested in MTT assays (**B**). Data are shown as a mean ± SEM of *n* = 3 independent experiments, each run in triplicate. * *p* < 0.05 vs. Ctrl; # *p* < 0.05 vs. HG alone in the respective conditions. Two-way ANOVA, followed by Tukey’s test.

Crystal violet assays were performed to compare the effects of 24, 48 and 72 h of treatment with VP13/126 or DMF in SIRC proliferation. After incubation of cells with increasing concentrations of VP13/126 (0.1, 1 and 5 μM) we observed a mild increase in cell proliferation after 24 h of treatment at 0.1 and 1 μM (*p* < 0.05) in comparison to control (Figure 3, Panel A). Similar effects were observed in DMF-treated cells; treatments at concentration of 10 μM for 24 h induced the proliferation rate of SIRC of about 15% (*p* < 0.05). As reported previously, crystal violet assays showed that exposure to HG reduced SIRC growth rate of about 25, 30 and 35% after 24, 48 and 72 h, respectively, (*p* < 0.05, Figure 3, Panel B), considered as sign of glucose-induced dysfunction [45]. Such a reduction in cell proliferation was not observed in HM-treated SIRC. Treatments for 24, 48 and 72 h with VP13/126 induced a significant improvement in cell proliferation of SIRC stimulated by HG, already at 1 and 5 μM concentration in comparison to controls (*p* < 0.05). On the other hand, DMF partially prevented the reduction of growth rate induced by HG; in fact, a significant restore was observed in SIRC co-treated with 25 μM of DMF for 72 h in comparison to control. Cell counts performed with Burker chambers did not detect any cell significant cell mortality. These data suggest that HG decrease cell proliferation in SIRC; this effect was better counteracted by 5 μM of VP13/126 than 25 μM of DMF.

### 3.5. VP13/126 Promoted Wound Healing in SIRC Cultured under High Glucose Conditions

**Figure 4:**
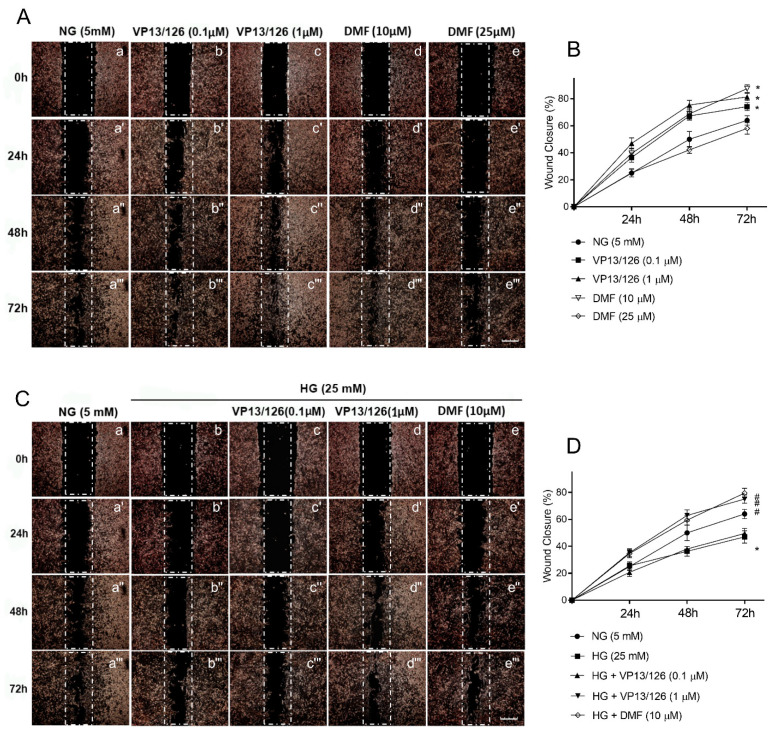
Promoting effects of VP13/126 on cell migration in SIRC challenged with high glucose (HG). (**A**): representative images of wound healing assays performed in normal glucose (NG) treated-cells with or without 0.1 and 1 µM of VP13/126 or 10 and 25 µM of DMF immediately after the scratch at 0 h (**a**–**e**), at 24 h (**a’**–**e’**), at 48 h (**a’’**–**e’’**) and at 72 h of incubation (**a’’’**–**e’’’**), respectively). (**B**): relative quantification of wound closure percentage carried out in NG conditions. (**C**): representative images of wound healing assays performed in NG cells, in HG-treated cells and in co-treated cells with HG plus 0.1, and 1 µM of VP13/126 and 10 µM of DMF immediately after the scratch at 0 h (**a**–**e**), at 24 h (**a’**–**e’**, respectively), at 48 h (**a’’**–**e’’**) and at 72 h of incubation (**a’’’**–**e’’’**, respectively). (**D**): relative quantification of wound closure percentage carried out in HG conditions. All images were acquired with a Leica microscope using a 20X magnification; scale bar = 200 µm. Data are shown as mean ± SEM of *n* = 3 independent experiments. * *p* < 0.05 vs. Ctrl; # *p* < 0.05 vs. HG alone in the respective conditions. Two-way ANOVA, followed by Tukey’s test.

Standard wound healing test was used in order to study the impact of our treatments in the wound repair SIRC capability. As shown in Figure 4 Panels A and B, the addition of 0.1 and 1 μM of VP13/126 in normal glucose conditions produced a significant increase in the wound closure rate of about 80 and 90% at 24 h, of about 35 and 40% at 48 h and of about 20 and 30% at 72 h, respectively, in comparison to control (*p* < 0.05). Regarding DMF, the treatment with 10 μM induced a significant (*p* < 0.05) increase of wound closure rate of about 80, 35 and 40% at 24, 48 and 72 h, respectively, in normal glucose conditions. Unlike VP13/126, the treatment with the higher dose of DMF (25 μM) produced no significant effect in the migration rate of SIRC compared to control. Cultures treated with HG showed a significant reduction of wound closure percentage producing a cell migration inhibition of about 40% at 48 h and 72 h after the beginning of the tests (Figure 4, Panels C and D). However, cell motility reduction elicited by HG was counteracted by 1 μM of VP13/126 and 10 μM of DMF, respectively. In fact, co-treatments with HG and 1 μM of VP13/126 or HG and 10 μM of DMF gave rise to an enhancement of the wound closure rate of about 40, 25 and 30% at 24, 48 and 72 h, respectively in comparison to cells treated in HG conditions (Figure 4, Panels C and D). No effects were observed with 0.1 μM VP13/126 in terms of wound healing in HG-treated SIRC (Figure 4). These findings clearly indicate that 1 μM of VP13/126 and 10 μM of DMF exert the same positive effects in counteracting the reduction of wound healing rate induced by HG in SIRC.

### 3.6. VP13/126 Induced Phospho- and Total-ERK1/2 as Well as HO-1 in SIRC Challenged with High Glucose

**Figure 5:**
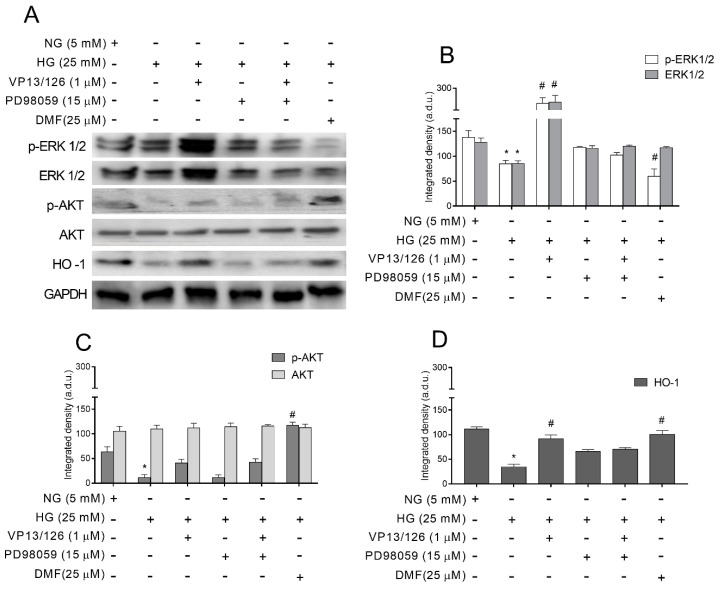
Effects of VP13/126 on phospho-ERK 1/2, total ERK1/2, phospho-AKT, total AKT and HO-1 protein expression in SIRC stimulated by high glucose. Proteins were analyzed by Western blot analysis in SIRC cultured in normal glucose (5 mM) or treated with HG (25 mM) alone or in presence of VP13/126 (5 µM), of PD98059 (15 µM), VP13/126 (5 µM) plus PD98059 (15 µM) or with 25 µM of DMF for 48 h. (**A**) shows representative images of immunoblot for phospho-ERK1/2 (p-ERK1/2), total ERK1/2 (ERK1/2), phospho-AKT (p-AKT), total AKT (AKT), heme oxygenase-1 (HO-1) and GAPDH proteins. GAPDH detection indicates the equal loading of 40 µg of protein in each lane. Immunoblot quantification regarding phospho- and total ERK1/2 (**B**), phospho- and total AKT (**C**) and HO-1 (**D**) protein levels were performed by densitometric analysis of each band (in arbitrary densitometry units, a.d.u.), through the Image J software. All data represent mean ± SEM obtained from at least three independent experiments. * *p* < 0.05 vs. Ctrl; # *p* < 0.05 vs. HG alone in the respective conditions. Two-way ANOVA, followed by Tukey’s test.

Previous experiments here reported indicated that VP13/126 exerts a more robust effect in the prevention of cell damage of SIRC after exposure to hyperglycemia and at lower concentrations than DMF. Therefore, we investigated the involvement of ERK1/2 and HO-1 in the protective effect of VP13/126 on cellular dysfunction induced by HG in SIRC. Phosphorylated amount of ERK1/2 (active form) was evaluated by Western blot in whole lysates of SIRC treated for 48 h with HG alone or in presence of VP13/126, of PD98059, the selective inhibitor of ERK1/2 phosphorylation, of these two compounds in combination or in presence of DMF. VP13/126 and DMF were tested at 5 and 25 μM of concentration, respectively, while PD98059 was tested at the subtoxic concentration of 15 μM. Moreover, we also verified whether the treatments were able to modulate the amount of phospho-AKT, described to mediate DMF antioxidant and anti-inflammatory effects [46]. As shown in Figure 5 (Panels A and B), treatments with HG significantly decreased the amount of phospho- and total ERK1/2 (of about 30 and 50%, respectively) as well as phospho-AKT (of about 75%, *p* < 0.05) and HO-1 (of about 70%, *p* < 0.05) protein levels. Instead, the co-treatments with HG and VP13/126 triggered a strong increase of both phospho- and total ERK1/2 beyond the control levels producing an enhancement of about 2-fold in comparison to the controls (*p* < 0.05).

Regarding phospho-AKT, HG and 5 μM of VP13/126 co-treatments produced a mild increase in its expression level of about 35% in comparison to HG-treated cells, whereas HO-1 expression increased up to control levels. Interestingly, no significant effect was produced following the adding of 5 μM PD98059 in HG conditions; however, it was able to completely prevent the induction of phospho-ERK1/2, total ERK1/2 and HO-1 when added in combination with HG and 5 μM of VP13/126. Moreover, phospho-AKT protein levels do not changed neither in cells co-treated with HG and 15 μM of PD98059 compared to HG conditions nor in cells co-treated with HG, 5 μM of VP13/126 and 15 μM of PD98059 compared to HG plus VP13/126-treated cells. All the treatments do not modify total AKT protein levels in comparison to control cells. In co-treated SIRC with HG and 25 μM of DMF we observed a significant reduction in phospho-ERK1/2 (of about 80%, *p* < 0.05) and increased levels of phospho-AKT and HO-1 protein levels in comparison to HG-treated cell, close to those observed in the controls. All together these data suggested that VP13/126 exerts a protective effect in HG-treated SIRC through a putative ERK1/2-mediated HO-1 activation mechanism.

### 3.7. ERK1/2 Mediated VP13/126-Induced Nrf2 Nuclear Translocation in SIRC Challenged by HG

**Figure 6:**
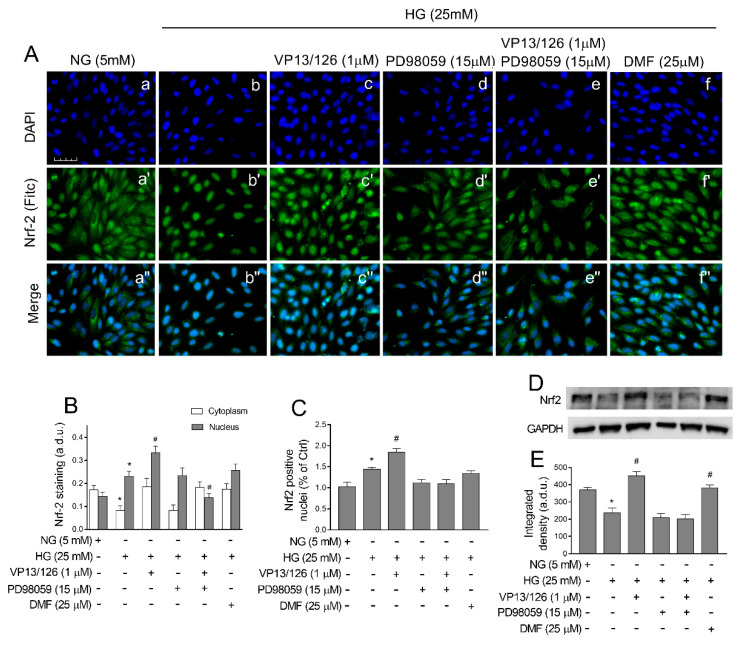
Nrf2 nuclear translocation and protein levels up-regulation in SIRC stimulated in high glucose in presence of VP13/126 or DMF. (**A**) shows representatives images of immunocytochemical stained for Nrf2 in SIRC cultured in normal glucose or in HG, (25 mM) without or with 1 µM VP13/126, 15 μM PD98059, VP13/126 plus PD98059 and 25 μM of DMF for 48 h (in green: a’–f’, respectively). Moreover, relative NucBlue-stained nuclei (a–f, respectively) and merged channels (a’’–f’’, respectively) are shown. All images were acquired with the Operetta High-Content Imaging System using a 20× magnification; scale bar = 100 µm. (**B**): high-content screening analysis was used to quantify green fluorescence (Fitc) relative to Nrf2 staining in cytoplasm and nuclei. (**C**): quantification of Nrf2 positive nuclei in SIRC stimulated with high glucose in presence of VP13/126 or DMF. (**D**): Western blot analysis of Nrf2 protein levels in whole lysates of SIRC treated with high glucose in presence of VP13/126 or DMF. GAPDH detection indicates the same loading of 40 μg of proteins in each lane. (**E**): immunoblot quantification through densitometric analysis of each band (in arbitrary density units, a. d. u.) performed by Image J software. Data are expressed as media ± SEM from at least six fields/well randomly selected and each reporting more than 15 cells/field. All data represent mean ± SEM obtained from at least three independent experiments. * *p* < 0.05 vs. control (Crtl); # *p* < 0.05 vs. HG. One-way ANOVA, followed by Tukey’s test.

As VP13/126 showed protective effects on SIRC treated with HG inducing the activation of ERK 1/2/HO-1 axis, we further confirmed these data by the analysis of its capability to stimulate Nrf2 nuclear translocation. High-content screening associated to immunofluorescence assays highlighted a basal level of immunoreactivity for Nrf2 protein (green fluorescence) in NG-cultured cells widespread both at cytoplasmic and nuclear level (Figure 6, Panel A, picture a’). Green fluorescence observed in HG-treated cells for 48 h was strongly reduced in the cytoplasm and was mostly localized in the nuclei (picture b’ vs. a’) where about 30% increase in immunoreactivity was detected in comparison to NG-cultured SIRC (Figure 5, panel A and B). The treatment with 5 μM of VP13/126 in presence of HG for 48 h restored Nrf2 immunoreactivity in the cytoplasm of SIRC to control levels (*p* < 0.05) enhancing the intensity of nuclear fluorescence of about 35% (*p* < 0.05) in comparison to HG-treated cells (picture c’ vs. b’). These results were confirmed by the evaluation of Nrf2 positive nuclei; in comparison to control, increased percentages of about 30 and 70% (*p* < 0.05) in positive nuclei were observed in SIRC treated with HG and HG plus VP13/126, respectively (Figure 6, Panel C). As reported in Figure 5 panels A and B, 15 μM PD98059 was able to prevent Nrf2 nuclear translocation induced by HG or by HG in combination with 5 μM VP13/126, as indicated by the reduction of green fluorescence intensity in SIRC treated in such conditions close to control levels (picture d’ and e’ vs. b’ and c’, respectively) in comparison to cells treated with HG plus 5 μM of VP13/126. Accordingly, percentage of Nrf2 positive nuclei was unchanged in SIRC stimulated with 15 μM of PD98059 in combination with HG or with HG plus VP13/126 in comparison to controls (Figure 6, Panel C). Green fluorescence intensity for Nrf2 was markedly higher in SIRC stimulated with HG plus 25 μM of DMF than in challenged cells with HG (pictures f’ vs. b’), although green fluorescence was observed particularly in the cytoplasmic region. According to immunocytochemical analysis, treatment with 25 μM of DMF in presence of HG provoked a modest increase in Nrf2 positive nuclei in comparison to HG-treated cells. In order to better analyze the VP13/126-mediated activation properties of Nrf2, we looked at its protein levels in whole lysate of SIRC. We observed that HG exposition of SIRC for 48 h reduced Nrf2 expression of about 60% (*p* < 0.05) in comparison to controls (Figure 6, panel D and E). Moreover, the treatment with 5 μM of VP13/126 in presence of HG both restored basal levels of Nrf2 protein levels and provoked about 40% (*p* < 0.05) increase in its levels in comparison to controls. As shown in Figure 6, Panels D and E, the presence of 15 μM of PD98059 do not produced any change in Nrf2 protein levels in comparison to HG-treated cells while completely prevented the VP13/126 mediated induction of Nrf2 in presence of HG. However, the treatments of SIRC with 25 μM of DMF in presence of HG for 48 h increased Nrf2 protein expression to control levels (*p* < 0.05). Overall, these findings support the detrimental role of HG in the protein levels of Nrf2 and suggest that VP13/126 mediates its nuclear translocation and up-regulation in an ERK1/2-dependent manner.

## 4. Discussion

Increased levels of oxidative stress are a critical factor in the onset of diabetic complications due to the adverse effects caused by glucose-induced ROS production [47]. The impairment of ROS scavenging capability induced by hyperglycemia represents one of the major causes of ROS accumulation and corneal epithelial cells dysfunctions [15,48]. High levels of glucose are shown to produce great changes in corneal physiology due to increased ROS production, such as a delay in corneal epithelial wound healing, the reduction of NAD^+^ biosynthesis, the attenuation of corneal sensation, mitochondrial dysfunction, the impairments of Akt, ERK1/2, epidermal growth factor receptor (EGFR) and Sirt1 activation, [49,50,51]. Efforts for the development of novel Nrf2/HO-1 pathway inducers are considered a promising molecular strategy for enhancing cellular response against oxidative stress [21,29]. We carried out a molecular docking study that guided the identification of best OH-1 inducer, simulating for small series of compounds (Scheme 2) the Michael addition to Cys151 of Keap1, the regulator of Nrf2 translocation and then of OH-1 expression [37]. VP13/126 had the best ΔG binding energy, compared to the other tested VP compounds and to DMF (Figure 1). In the present study, we show that the detrimental role of high glucose produced effects in SIRC according to literature such as the reduction of cell viability, proliferation rate and wound healing capability (Figure 2, Figure 3 and Figure 4) [14,52,53]. Regarding the molecular mechanisms, we showed that 48 h of treatment with HG reduced both ERK1/2 and Akt signaling, as indicated by the reduced levels of phosphorylated form of these proteins [14,15,25]. VP13/126 was able to exert a considerable protective effect towards high glucose concentrations; in fact, this compound restored such cellular functions in HG-stimulated SIRC. In particular, we observed that 1 μM VP13/126 produced a more robust effects compared to those obtained using DMF tested at 25 μM. The presence of 1 μM VP13/126 mediated a more prolonged effect in the recovery of cell viability than DMF in HG-treated SIRC as indicated by MTT value similar to control in co-treated cells up to 72 h (Figure 2). Instead, DMF was able to enhance SIRC viability in presence of HG considerably at the concentration of 25 μM, although this effect was lost after 24–48 h of co-treatment. Moreover, VP13/126 showed a greater effectiveness in the prevention of HG-induced reduction of the SIRC proliferation rate than DMF; in fact, as reported in Figure 3, a significant recovery in the proliferation of HG-stimulated SIRC was mediated by VP13/126 starting from 24 h of co-treatment at a concentration of 1 μM. Equimolar concentration of DMF produced a more delayed effect only after 72 h of co-treatment in presence of HG. The protective effect of VP13/126 was highlighted also regarding cell motility in SIRC treated with HG; as shown in Figure 4, 1 μM of VP13/126 and 10 μM DMF triggered similar increase in SIRC wound closure both in normal and in HG conditions. Interestingly, high amount of DMF (25 μM) were partially active in the prevention of HG-induced cell viability reduction in SIRC but was unable to counteract the reduction of cell proliferation and motility promoted by HG concentrations. Overall, these data suggest that VP13/126 prevents HG-induced damage, showing a greater effectiveness in ERK1/2 activation than DMF as demonstrated by the strong increase in phospho-ERK1/2 protein levels in SIRC (Figure 5). Western blot data indicate that 5 μM of VP13/126 also produced a new synthesis of ERK1/2 as indicated by increased protein levels detected by anti-total ERK1/2. The involvement of ERK signaling in glucose-induced ocular disease is widely described in the literature [54,55,56,57]. However, ERK1/2 phosphorylation and its associated activation was shown to protect skin cells against oxidative stress by promoting the up-regulation of Nrf2 and its downstream regulated genes [58]. Moreover, it was demonstrated the essential role of ERK 1/2 activation in the Nrf2 nuclear translocation as it mediates the phosphorylation of its amino acid residue Ser40 [59]. Our findings indicate that VP13/126 exerts a protective effect in HG-treated SIRC through an ERK1/2-mediated HO-1 activation mechanism. In our study, we correlate ERK1/2 phosphorylation to Nrf2/HO-1 axis activation using the selective ERK1/2 inhibitor PD98059. As validated in previous studies, PD98059 was able to prevent ERK1/2 phosphorylation in our model (Figure 4); in such a condition, we detected a strong reduction of VP13/126 capability to induce Nrf2 translocation and HO-1 up-regulation (Figure 5 and Figure 6) [24,60,61]. Our results confirm the upstream involvement of ERK1/2 signaling with respect to the VP13/126-induced activation of Nrf2 in HG-stimulated SIRC.

In SIRC treated with HG and 25 μM DMF we observed a significant increase of HO-1 protein levels in comparison to HG-treated cell, close to those observed in the controls. In our model, DMF seems to promote a partial translocation of Nrf2 through an Akt-dependent mechanism. Considering the aim of our study, we do not correlate the increase of phospho-AKT protein levels to HO-1 up-regulation nor we included data regarding the effects of PD98059 in co-treated SIRC with DMF and high glucose, as we included DMF as positive control. However, this data is certainly in agreement with the HO-1 inducer activity of DMF mediated by phospho-AKT and observed in other models [35,62,63]. Reported data indicate that different compounds induce Nrf2/HO-1 axis in hyperglycemia-stimulated epithelial cells through the involvement of both Akt and ERK 1/2 signaling [15,64].

In this study, the greater efficacy of VP13/126 than DMF against HG-induced damage in SIRC was related to its strong activation of the ERK pathway rather than Akt. Moreover, our in silico data indicate that this compound induce Nrf2 nuclear accumulation and HO-1 activation also through a Keap1-dependent mechanism. Covalent docking, in fact, revealed a stronger binding affinity of VP13/126 than DMF to Keap1 homodimer. VP13/126 binding results in the destabilization of Keap1 homodimer by increasing its electrophile properties and, in turn, by promoting Nrf2 translocation to the nucleus [65]. Compared to DMF, the greater effectiveness of VP13/126 in the prevention of cellular damage in HG-stimulated SIRC seems to be attributed to its capability to activate Nrf2 via a Keap1-dependent and Keap1-independent mechanisms. Our findings are in accordance with the idea that Nrf2 activation is mediated by a coupling of a direct mechanism Keap-1-dependent with an indirect mechanism ERK1/2 phosphorylation-dependent. It was recently reported that antioxidant activity can be partially attributed to induction of HO-1, by a Keap1-independent Nrf2 activation mechanism based on ERK phosphorylation [66].

## 5. Conclusions

Corneal epithelial dysfunction is associated to inflammatory conditions or ocular surface disease, producing an impairment of corneal re-epithelialization. Diabetes causes pathological ocular changes elicited by a chronic oxidative stress and an inflammatory status. Here, we analyzed the putative role of a new compound VP13/126, a DMF derivative, to protect SIRC from cell damage induced by HG damage. Our data suggested that VP13/126 prevents HG-induced SIRC damage more than DMF, showing a greater effectiveness in ERK1/2 phosphorylation/activation. Moreover, we produced findings supporting the pivotal role of ERK1/2 in the activation of the pro-survival Nrf2/HO-1 axis using the selective ERK1/2 inhibitor PD98059. High-content screening associated to immunofluorescence assays was used to highlight that VP13/126 mediates Nfr2 nuclear translocation and up-regulation in an ERK1/2-dependent manner, with a greater effectiveness than DMF. Our in silico data suggested that VP13/126 induces Nrf2 nuclear translocation and the consequent HO-1 activation also through a Keap1-dependent mechanism. Covalent docking, in fact, revealed a stronger binding affinity of VP13/126 than DMF to Keap1 homodimer, resulting in the destabilization of Keap1 homodimer by increasing its electrophile properties. These results confirmed the role of Nrf2/HO-1 pathway as a therapeutic target for the treatment of the corneal re-epithelialization impaired by hyperglycemia.

## Data Availability

The data presented in this study are available on request from the corresponding author. The data are not publicly available due to reasons of privacy.
